# HOPS and Vps13 Have Antagonistic Roles in Regulating Lipid Droplet Morphology

**DOI:** 10.3390/cells15191724

**Published:** 2026-09-22

**Authors:** Jie Wang, Saidaiguli Abulimiti, Jingping Chen, Xinping Zheng, Wanjing Chen, Yang Lei, Xingya Xu

**Affiliations:** Institute of Future Agriculture, Northwest A&F University, Yangling 712100, China; wangjie0202@nwafu.edu.cn (J.W.); 990917@nwafu.edu.cn (S.A.); 2025051345@nwafu.edu.cn (J.C.); zxp2025@nwafu.edu.cn (X.Z.); linsiran@nwafu.edu.cn (W.C.); 2023060716@nwafu.edu.cn (Y.L.)

**Keywords:** fission yeast, lipid droplet, Erg6, HOPS complex, Vps1302

## Abstract

Lipid droplets are dynamic organelles that store neutral lipids (mostly triacylglycerols and sterol esters) and are bound by a single phospholipid monolayer. The formation and breakdown of the droplets are important for cellular metabolism; however, the molecular mechanisms regulating their biogenesis and turnover are still obscure. In this study, we used Erg6-yeGFP as a lipid droplet marker, isolated 1100 temperature-sensitive mutants, and observed lipid droplet morphology in these mutants by fluorescence microscopy. Six mutants with abnormal lipid droplet morphology were obtained and next-generation sequencing identified responsible mutations in genes encoding subunits of HOPS complex or its binding partner Ypt7. Therefore, the genetic screen indicated that the HOPS complex plays a critical role in maintaining lipid droplet morphology. In the absence of Vps11, vacuoles were fragmented with dot-like structures and degradation of lipid droplet surface protein Erg6 (under glucose starvation condition) decreased dramatically. A genetic screen for mutations that could rescue the abnormal lipid droplet morphology in *Δvps11* by fluorescence microscopy identified two independent mutations in *vps1302*. Vps1301 and Vps1302 are paralogs that function as intermembrane lipid transfer proteins. Deletion of either *vps1301* or *vps1302* rescued the abnormal lipid droplet morphology in *Δvps11* as well.

## 1. Introduction

Eukaryotic cells store neutral lipids, primarily triacylglycerols and sterol esters, within specialized dynamic organelles termed lipid droplets (LDs). Unlike canonical double-membrane organelles, LDs possess a hydrophobic core surrounded by a single phospholipid monolayer. This monolayer harbors a characteristic set of integral and peripheral proteins, among which Erg6 is one of the most abundant LD-resident proteins in yeast, that collectively control LD formation, growth, and catabolism [1,2]. Beyond serving as lipid reservoirs, LDs act as multifunctional signaling hubs that impinge on membrane trafficking, protein quality control, and cellular stress adaptation. Disrupted LD dynamics and aberrant morphology are associated with a wide range of human pathologies, including obesity, hepatic steatosis, and neurodegenerative diseases [3]. Although research in recent decades has greatly expanded our knowledge of LD biology, the complete set of cellular factors that dictate LD shape and couple LD behavior to the function of other organelles has yet to be fully mapped.

Two major pathways drive LD turnover in eukaryotes. The first is cytosolic lipolysis, in which endogenous lipases directly hydrolyze stored neutral lipids. The second is lipophagy, a selective form of autophagy that targets LDs to lysosomes (equivalent to vacuoles in yeast) for degradation by luminal hydrolase [4,5]. In *Schizosaccharomyces pombe*, glucose deprivation is a robust trigger of lipophagy. This process can be measured either through observing the engulfment of LDs into the vacuole or via a GFP cleavage assay, in which the LD-localized marker Erg6-yeGFP is processed by vacuolar proteases to release a stable free GFP fragment [6,7]. While the core autophagy (Atg) machinery is known to be essential for lipophagy, upstream regulatory factors that link vacuolar membrane fusion, organelle structural integrity, and LD morphogenesis are only beginning to be identified.

The homotypic fusion and vacuole protein sorting (HOPS) complex is a multisubunit tethering factor conserved across eukaryotes, with well-documented roles in membrane fusion along the late endocytic and autophagic pathways. The Mon1–Ccz1 complex serves as the guanine nucleotide exchange factor for the Rab GTPase homolog Ypt7, the yeast homolog of mammalian Rab7. Upon Ypt7 activation, the HOPS complex acts as a Ypt7 effector and multisubunit tethering factor that promotes tethering and subsequent fusion of late endosomes and autophagosomes with vacuoles [8,9]. Studies in budding yeast have shown that deletion of any single HOPS subunit leads to severe vacuolar fragmentation and defective autophagic degradation [10,11]. Bouchez et al. previously demonstrated in the budding yeast *Saccharomyces cerevisiae* that both Ypt7 and the HOPS complex are required for normal LD morphology and that loss of HOPS function results in aberrant LD clustering and morphological defects [12]. Despite this extensive characterization of HOPS in vacuole biology, whether the complex influences LD morphology and lipophagic flux in fission yeast remains unaddressed [13].

Lipid transfer proteins catalyze non-vesicular lipid movement at inter-organelle membrane contact sites, and a growing body of work implicates these factors as key regulators of LD biogenesis and inter-organelle communication [14]. The Vps13 family represents an evolutionarily conserved class of intermembrane lipid transporters that mediate bulk lipid transfer between adjacent membrane compartments [15,16]. The *S. pombe* genome encodes two Vps13 paralogs, Vps1301 and Vps1302. To date, however, their physiological functions in LD homeostasis and whether they interact functionally with the HOPS complex have not been examined.

In this work, we carried out an unbiased forward genetic screen for temperature-sensitive mutants in fission yeast, using Erg6-yeGFP as a fluorescent LD marker. From this screen, we identified mutations in genes encoding HOPS complex subunits and their interacting partner Ypt7 as causes of aberrant LD morphology. Deletion of *vps11*, a core HOPS subunit, resulted in widespread vacuolar fragmentation in rich YES medium and a complete block of the degradation of LD surface protein (Erg6) under glucose starvation condition; however, genetic analyses indicated that the LD morphological defect occurs independently of lipophagic impairment. A suppressor screen based on temperature sensitivity found that deletion of *ins1* partially rescues the growth defect of *Δvps11* cells at restrictive temperature but does not restore normal LD morphology. In parallel, an LD morphology-based suppressor screen identified loss-of-function mutations in *vps1302*, as well as deletion of either *vps1301* or *vps1302*, as suppressors of the *Δvps11* LD phenotype. Together, these findings reveal a previously unrecognized, lipophagy-independent function of the HOPS complex in LD shape control, and define a genetic interaction between HOPS-dependent vacuolar integrity and Vps13-mediated lipid transport in regulating LD morphology.

## 2. Materials and Methods

### 2.1. Media and Reagents

YPD plates were used to grow *S. pombe* strains and YES medium was used to culture *S. pombe* strains for microscopic observation. YES medium with 0.04% glucose (standard YES with the glucose concentration was reduced from 20 g/L to 0.4 g/L) was used to induce autophagy for immunoblotting. MEA plates were used for germinating fungal spores. Nile red (C2051S, Beyotime Biotechnology) was used to stain cellular lipids.

### 2.2. Collection of Ts Mutants

A wild-type strain containing *erg6-yeGFP* was used for mutagenesis. Log-phase cells cultured in YPD medium were collected and suspended in 50 mM TM buffer (Tris-Maleate solution, pH 6.0). Cells were treated with 150 μg/mL N-methyl-N’-nitro-N-nitrosoguanidine (NTG, purchased from Aladdin Scientific (Shanghai, China) with catalog M105583) at 30 °C for 30 min. NTG-treated cells were collected and cultured in YPD medium for 8 h. A total of 150 YPD plates were prepared with 1 × 10^4^ cells spread on each of them. The YPD plates were cultured at 30 °C for 3 days and colonies were copied onto new YPD plates using replica plating technique. One copy of the plates was cultured at 30 °C and the other copy of the plates was cultured at 37 °C. Strains that could grow at 30 °C but could not grow at 37 °C were collected as ts mutants. Temperature sensitivity of the strains was verified by streaking and then the strains were stored in a −80 °C fridge.

### 2.3. Mutagenesis in the Δvps11 erg6-yeGFP Mutant

The *Δvps11 erg6-yeGFP* mutant strain was used for mutagenesis. The mutagenesis procedure followed the one described above with modifications. After NTG treatment, cells were cultured in YPD medium for 6 h. A total of 10 YPD plates were prepared with 1 × 10^4^ cells spread on each of them. The YPD plates were cultured at 30 °C for 3 days. Colonies were picked up and cultured in YES medium at 30 °C overnight. Lipid droplet morphology in the mutants (indicated by Erg6-yeGFP) was observed by fluorescence microscopy.

### 2.4. Construction of Deletion Mutants

The 972 *h-* wild-type strain and the *h+* wild-type strain were used as the host strains for constructing the deletion mutants. The targeting vectors were constructed based on the pBluescript plasmid containing either a hygromycin-resistant antibiotic marker (*hphMAX6*) or a G-418-resistant antibiotic marker (*kanMAX6*). A ~500 bp sequence upstream of the target gene was amplified by PCR and ligated into the vector upstream of the antibiotic gene and a ~500 bp sequence downstream of the target gene was amplified by PCR and ligated into the vector downstream of the antibiotic gene. The targeting vectors were linearized and transformed into the host strains and cells were spread on YPD plates containing corresponding antibiotic and cultured at 30 °C for 4 days. Colonies were picked up and mutants were verified by PCR and sequencing. Hygromycin B was purchased from Meilunbio (Shanghai, China) (MB6158) and G-418 Sulfate was purchased from Coolaber (Beijing, China) (CG5471).

### 2.5. Phenotypic Observation

Cells were cultured to 0.6~1 × 10^7^ cells per mL in YES medium; then, cells were enriched by centrifugation and observed under a spinning disk confocal system (Nikon CSU-W1). The number of Erg6-yeGFP dot in each cell in the microscopic images was counted manually and the percentage of cells having more than 3 Erg6-yeGFP dots was calculated by counting the number of cells qualified in 200 cells.

### 2.6. Next-Generation Sequencing and Mutation Identification

Next-generation sequencing and mutation identification followed the protocols described in our previous publications [17,18,19]. *Schizosaccharomyces pombe* reference genome assembly ASM294v3 was used as reference for data mapping.

Since all the F1 off-springs having the expected phenotypes were mixed together as one sample for next-generation sequencing, the responsible mutations should appear with 100% frequency; therefore, mutations with 100% frequency (or very close to 100%), identified from the next-generation sequencing results, were selected as the responsible mutations.

## 3. Results

### 3.1. A Genetic Screen for Mutants with Abnormal Lipid Droplet Morphology

Lipid droplets have a hydrophobic core of neutral lipids that is surrounded by a phospholipid monolayer [3], with a distinct cohort of integral and peripheral proteins (such as Erg1 and Erg6) that govern their biogenesis, growth and turnover [20,21]. Among the proteins bound to the lipid droplets, Erg6 is one of the most abundant proteins [22]. Erg6 has been widely used as a reliable fluorescent marker for visualizing LD morphology in both budding and fission yeast. Its localization is highly specific to LDs, and endogenous tagging does not alter LD biogenesis or cellular lipid composition [5,22]. Therefore, we used Erg6-yeGFP as an indicator of lipid droplet.

Temperature-sensitive mutants (which can grow at permissive temperature but cannot grow at restrictive temperature) provide us a way to study functions of essential genes in budding yeast and fission yeast. In order to identify genes (especially essential genes) whose protein products regulate lipid droplet, we introduced random mutations into the wild-type strain (containing *erg6-yeGFP*) by treating the cells with N-methyl-N’-nitro-N-nitrosoguanidine (NTG or MNNG), a potent alkylating agent that was widely utilized as a chemical mutagen. After screening ~30,000 colonies, 1100 ts mutants (that could form a colony at 30 °C but could not form a colony at 37 °C) were obtained. Then, the morphology of lipid droplets (indicated by Erg6-yeGFP signals) in these mutants was detected by fluorescence microscopy one by one after incubation at restrictive temperature 37 °C for 2 h (Figure 1A). Finally, abnormal lipid droplet morphology (indicated by diffused Erg6-yeGFP signals) was observed in six mutants (*LD-38*, *LD-126*, *LD-395*, *LD-544*, *LD-638*, and *LD-911*). Later, we realized that the abnormal lipid droplet morphology observed in the mutants still remained when cultured at the permissive temperature (30 °C) (Figure 1B). Therefore, permissive temperature (30 °C) was used to observe lipid droplet morphology in these mutants in the following experiments.

### 3.2. Mutations in HOPS Complex Genes Caused Abnormal Lipid Droplet Morphology

To identify the responsible mutations in the six mutants that exhibited abnormal lipid droplet morphology, we took two complementary strategies: (1) the mutants were backcrossed with the wild-type strain (containing *erg6-yeGFP*), all the F1 off-springs (around 64 off-springs) were observed by fluorescence microscopy and all the off-springs with abnormal lipid droplet morphology were mixed together in equal cell number and then used as materials for next-generation sequencing [23] (Tables S1–S5); (2) the mutants were backcrossed with the wild-type strain (containing *erg6-yeGFP*), the off-springs were observed by fluorescence microscopy and one off-spring with abnormal lipid droplet morphology was selected for next round of backcross with the wild-type strain (containing *erg6-yeGFP*), five rounds of backcrosses were performed for each of the six mutants to remove silent mutations in the original strains and next-generation sequencing was performed for the six mutants (Tables S6–S9). By analyzing the mutations identified from the next-generation sequencing results and comparing the mutations identified in the two strategies described above, the responsible mutations of the abnormal lipid droplet morphology in the mutants were determined. Mutations in genes encoding subunits of the HOPS complex or its interacting partner Ypt7 were identified in five of the six mutants (Vps41-W479S mutation was detected in *LD-638*, but further verification was required to assign it as the causal allele) (Figure 1C). Specifically, *LD-38*, *LD-126*, *LD-395*, and *LD-544* carry premature stop codons in *vam6* or *vps11*, respectively, all of which encode core structural subunits of the hexameric HOPS tethering complex [24]. The remaining *LD-911* has a mutation in *ypt7*, which encodes the Rab7 GTPase homolog that recruits and activates the HOPS complex on endosomal and vacuolar membranes [9]. Although these genes (*vam6*, *vps11*, and *ypt7*) were not essential, mutations in these genes or deletion of these genes caused temperature sensitivity [25,26].

Deletion mutants of *vps11* or *ypt7* were then constructed in the wild-type background and the abnormal lipid droplet morphology was obvious in these mutants too (all cells in wild type have one to three Erg6-yeGFP dots and 80% of them have two dots; however, 81% and 85% of cells of *Δvps11* and *Δypt7* contain more than three Erg6-yeGFP dots, respectively), therefore confirming that the HOPS complex and its interacting partner Ypt7 are essential for maintaining lipid droplet morphology (Figure 2).

### 3.3. Vacuolar Fragmentation and Protein Degradation Defects in HOPS Mutants

It was reported that in the absence of Vps41, a subunit of the HOPS complex, vacuoles were highly fragmented with several dot-like structures [13]. We observed vacuolar morphology using Cpy1-mCherry as an indicator (Cpy1 locates in vacuole lumen) in the HOPS mutants and found that Cpy1-mCherry signals typically appeared as 1–3 large, round vacuoles in wild-type cells, whereas they were much more diffused in the cytoplasm with dot-like structures in the HOPS mutants (Figure 3A).

A major function of the HOPS complex is to mediate autophagosome–vacuole fusion, a required step for macroautophagy and lipophagy [27]. Nutrient starvation induces lipophagy, which is a process of selective degradation of lipid droplets through autophagy. This process can be studied in yeast via a GFP cleavage assay: when LDs are delivered to the vacuole through lipophagy, the LD-resident Erg6-yeGFP is processed by vacuolar proteases, releasing a stable, protease-resistant free GFP fragment that can be detected by immunoblotting [5,28,29]. The degradation of LD surface protein Erg6 was detected in wild type after glucose starvation (glucose concentration in the YES medium was reduced from 2% to 0.04%) as a free GFP band (~25 kDa) in immunoblotting; however, the free GFP band was not detected in the *Δvps11* mutant, indicating that degradation of LD surface protein Erg6 was blocked in the absence of Vps11 (Figure 3B). Similarly, the free GFP band could not be detected in autophagy mutants (*Δatg5*, *Δatg8*, and *Δatg15*) (Figure 3C).

A central question arising from these observations is whether the LD morphological defects in the HOPS mutants are related to impaired lipophagy. To address this, we examined LD morphology in core autophagy mutants. The lipid droplet morphology in the mutants of macroautophagy machinery (*Δatg5* and *Δatg8*) in rich YES medium looked similar to those in wild type and all cells counted contain one to three Erg6-yeGFP dots as those in wild type (Figure 3D), and introduction of mutants of macroautophagy machinery (*Δatg5* and *Δatg8*) into *Δvps11* did not affect the abnormal lipid droplet morphology observed in the *Δvps11* single mutant in rich YES medium, as 85% and 87% cells of *Δvps11 Δatg5* and *Δvps11 Δatg8*, respectively, have more than three Erg6-yeGFP dots which are comparable to that in the *Δvps11* single mutant (whether the mutants of macroautophagy machinery have an effect on lipid droplet morphology under glucose starvation condition is not investigated in this study) (Figure S1). To further address this question, we examined the degradation of LD surface protein Erg6 in mutants lacking components of the endosomal sorting complex required for transport (ESCRT) pathway. The ESCRT machinery mediates sorting of ubiquitinated membrane proteins at late endosomes and has previously been implicated in lipophagy [7,30]. We found that the free GFP bands in the mutants (*Δsst2*, *Δsst4*, and *Δsst6*) of the endosomal sorting complexes required for transport (ESCRTs) were indistinguishable from that in the wild type after glucose starvation (Figure 3E). In addition, the lipid droplet morphology in the ESCRT mutants (*Δsst2*, *Δsst4*, and *Δsst6*) in rich YES medium was similar to that in wild type as all cells have one to three Erg6-yeGFP dots (Figure S2A). Addition of ESCRT pathway mutants (*Δsst2*, *Δsst4*, and *Δsst6*) into *Δvps11* did not affect the abnormal lipid droplet morphology in rich YES medium at all, as 79%, 81%, and 81% of cells of *Δvps11 Δsst2, Δvps11 Δsst4* and *Δvps11 Δsst6* double mutants, respectively, have more than three Erg6-yeGFP dots which are comparable to that in the *Δvps11* single mutant (Figure S2B).

### 3.4. Deletion of ins1 Rescued the Temperature Sensitivity of Δvps11 but Not the Abnormal Lipid Droplet Morphology

The ts mutants could not form colony at restrictive temperature. To identify pathways regulating Vps11, we performed a suppressor screen for mutations that could rescue the temperature sensitivity of *LD-395* (responsible mutation: Vps11-Q825Stop) at restrictive temperature. Cells of the *LD-395* mutant were spread on the rich YPD agar plates and incubated at 35 °C for 4 days and colonies (revertants) were collected for next-generation sequencing. Mutations in *ins1*, which is involved in regulation of sterol biosynthesis [31], were identified by next-generation sequencing followed by bioinformatical analysis (Figure 4A). Mutations in *sck1* were identified too, but they were not selected for further analysis, as mutations in *sck1* were reported as general suppressors of high temperature [32]. To confirm the genetic suppression, we constructed a *Δvps11 Δins1* double deletion mutant and tested its growth across a range of temperatures. Deletion of *ins1* rescued the temperature sensitivity of the *Δvps11* mutant partially at restrictive temperatures (33–37 °C) (Figure 4B). Then we observed lipid droplet morphology in the *Δvps11 Δins1* double mutant. In total, 84% of cells of *Δvps11 Δins1* contain more than three Erg6-yeGFP dots, which is comparable with that in *Δvps11*; therefore, the abnormal lipid droplet morphology introduced by *Δvps11* was not rescued by *Δins1* (Figure 4C). This result was confirmed by Nile Red staining of neutral lipids, ruling out the possibility that the observed phenotype was an artifact of altered Erg6-yeGFP expression or localization.

### 3.5. Mutations in Vps1302 Suppressed the Abnormal Lipid Droplet Morphology in Δvps11

Since the growth-based suppressor screen failed to identify the mutations that could suppress the abnormal lipid droplet morphology in the *Δvps11* mutant, we took a visual observation strategy here. To identify specific regulators of LD morphology acting downstream of or in parallel with HOPS, a genetic screen for mutations that could suppress the abnormal lipid droplet morphology in the *Δvps11* mutant was performed by observing Erg6-yeGFP signals by fluorescence microscopy (Figure 5A). First, random mutations were introduced into the *Δvps11* mutant (containing *erg6-yeGFP*) by treating the cells with N-methyl-N’-nitro-N-nitrosoguanidine (NTG or MNNG). A total of 960 colonies formed on the rich YPD agar plates were picked up and cultured in rich YES medium, a fluorescent microscopy was then applied to observe the lipid droplet morphology (indicated by Erg6-yeGFP signals) in these mutants one by one. Two mutants with normal lipid droplet morphology (*LDR-57* and *LDR-254*) were identified from the 960 mutants (62% cells of each of the two mutants contain one to three Erg6-yeGFP dots) (Figure 5B). Then, the *LDR-57* and *LDR-254* mutants were backcrossed with their host strain (*Δvps11 erg6-yeGFP*), 64 F1 generation off-springs for each mutant were observed by fluorescence microscopy, the off-springs of same mutant with wild-type-like Erg6-yeGFP signals were mixed together as one sample for next-generation-sequencing analysis. Finally, mutations in *vps1302* (Vps1302-Q2447Stop in *LDR-57* and Vps1302-Q1660Stop in *LDR-254*) were identified as the responsible mutations that could suppress the abnormal lipid droplet morphology in the *Δvps11* mutant (Figure 5C, Tables S10 and S11).

Vps13 family proteins are evolutionarily conserved lipid transporters that localize to inter-organelle membrane contact sites and mediate non-vesicular bulk lipid transfer between adjacent membrane compartments [15,16]. Vps1302 has a paralog named Vps1301 in fission yeast. To confirm the genetic suppression of the abnormal lipid droplet morphology in the *Δvps11* mutant, we generated single deletion strains of *vps1301* and *vps1302* and then introduced each of them into the *Δvps11* mutant using the tetrad dissection technology. Consistent with the genetic suppressor screen results, deletion of either *vps1301* or *vps1302* suppressed the abnormal lipid droplet morphology in the *Δvps11* mutant (70% and 73% cells of the *Δvps11 Δvps1301* and *Δvps11 Δvps1302*, respectively, have one to three Erg6-yeGFP dots) (Figure 6A and Figure S3). We also detected the degradation of LD surface protein Erg6 in the *LDR-57* and *LDR-254* by immunoblot detection of the free GFP band after glucose starvation. Immunoblot analysis showed that, similar to *Δvps11* single mutants, LDR-57 and LDR-254 double mutant cells still lacked detectable free GFP under glucose starvation conditions (Figure 6B). Thus, the defect of the degradation of Erg6 observed in *Δvps11* was not recovered in the *LDR-57* and *LDR-254* mutants.

## 4. Discussion

Through forward genetic screens in fission yeast, we demonstrate here that the HOPS tethering complex plays a previously unappreciated role in controlling LD morphology. In contrast to mammalian high-throughput screening platforms, which depend on expensive CRISPR libraries, automated high-content imaging systems and large volumes of specialized reagents, the chemical mutagenesis-based phenotypic screening strategy adopted in this study carries a distinct cost advantage. With Erg6-yeGFP as a straightforward fluorescent readout, this approach enables unbiased, large-scale screening of morphological phenotypes at low experimental cost and requires no sophisticated precision instrumentation or costly commercial assay kits. Using this cost-effective genetic system, we found that disruption of HOPS subunits, or of their Rab GTPase partner Ypt7, consistently led to abnormal LD structure, along with pronounced vacuolar fragmentation and defective degradation of LD surface protein Erg6. Our findings in the fission yeast *Schizosaccharomyces pombe* aligned well with a previous report in the budding yeast *Saccharomyces cerevisiae* [12]. Using two independent suppressor screens, we further show that the temperature-sensitive growth phenotype of *Δvps11* can be uncoupled from its LD morphological defect. In particular, loss of the lipid transfer protein Vps1302 specifically suppresses LD abnormalities in *Δvps11* cells, without restoring the degradation of LD surface protein. Collectively, these data indicate that the HOPS complex controls LD morphology via a mechanism genetically distinct from its function in autophagy and that Vps13-mediated lipid transport acts antagonistically to HOPS in this process.

Our finding that HOPS dysfunction disrupts both vacuolar morphology in rich medium and the degradation of LD surface protein under glucose starvation condition is consistent with extensive prior work in budding yeast, where HOPS is required for vacuole homotypic fusion and autophagosome-vacuole docking [8,9]. As a conserved multisubunit tethering complex, HOPS interacts with SNARE machinery to drive membrane fusion events in the late endocytic and autophagic pathways, a function preserved from yeast to mammalian cells [9,27]. The LD morphological defects observed in HOPS mutants may not arise as a secondary effect of impaired lipophagy and several independent observations support it. First, although the degradation of LD surface protein was blocked in the core autophagy mutants after glucose starvation, deletion of the core autophagy genes could not rescue the abnormal lipid droplet morphology in the *Δvps11* mutant in rich YES medium. Second, *vps1302* suppressor mutations fully restored the abnormal LD shape in *vps11* cells to similar to that in wild type but did not recover the defects of the degradation of LD surface protein observed in the *Δvps11* mutant after glucose starvation. Third, mutants lacking components of the ESCRT complex, which mediates endosomal sorting, displayed normal degradation of LD surface protein Erg6 as that in wild type under glucose starvation. However, it was reported in budding yeast that the degradation of LD surface proteins relies on autophagy and can occur independently of lipophagy, as after glucose restriction internalization of LDs into the vacuole was severely blocked in the ESCRTs mutants but the degradation of LD surface proteins (Erg6, Faa4) was not blocked [28]. To further address the potential effects of lipophagy on regulating lipid droplet morphology, internalization of LDs into the vacuole should be measured in the mutants.

From our suppressor screen of temperature sensitivity, we identified mutations in *ins1*, a gene implicated in sterol biosynthesis, as partial suppressors of the *Δvps11* growth defect at restrictive temperature. Importantly, however, *ins1* deletion failed to rescue the abnormal LD morphology caused by loss of Vps11. This clear phenotypic uncoupling indicates that the temperature-sensitive growth defect and the LD morphological defect of HOPS mutants arise via distinct downstream mechanisms. This finding also bears physiological significance: as the *ins1* mutation rescues the growth defect resulting from *vps11* deletion but not the LD morphology defect, proper LD morphology does not appear to be strictly required for cell viability under standard growth conditions. Given the function of Ins1 in sterol biosynthesis, one plausible explanation is that altered membrane sterol content partially alleviates the membrane fusion defects caused by HOPS deficiency, thereby improving cell viability, but does not re-establish the vacuole–LD contacts necessary for normal LD structure. Ergosterol, the major sterol in yeast, modulates membrane separation and homotypic fusion efficiency [33,34]; thus, changes in sterol composition could partially compensate for impaired tethering complex function. This observation further supports the specificity of the genetic interaction between HOPS and Vps13 in LD regulation.

Our genetic data identify that Vps1302 as a suppressor of the *Δvps11* LD phenotype, along with its paralog Vps1301, supports a working model in which Vps13-mediated lipid transport drives LD morphological defects when vacuolar integrity is disrupted. Vps13 family proteins are lipid transporters that localize to multiple inter-organelle contact sites and mediate bulk lipid exchange between closely apposed membrane compartments [15,16]. Biochemical studies have demonstrated that Vps13 proteins can transfer multiple lipid species including glycerophospholipids and sterols and are critical for maintaining membrane lipid homeostasis at organelle interfaces [35]. We propose that, in wild-type cells, intact vacuoles form stable contact sites with LDs, and Vps13-dependent lipid exchange at these interfaces is tightly controlled to preserve normal LD size and morphology. When HOPS function is lost, vacuolar fragmentation disrupts these contact sites and rewires lipid flux between the two organelles. In this context, unregulated Vps13-mediated lipid transfer leads to aberrant lipid accumulation at LDs, which in turn gives rise to morphological abnormalities. Deletion of either *vps1301* or *vps1302* reduces this misdirected lipid flux and, in doing so, restores normal LD morphology. Since lipophagy strictly depends on HOPS-mediated autophagosome–vacuole fusion, a step that cannot be bypassed by altering lipid transport, loss of Vps13 proteins does not restore lipophagic activity.

From a methodological standpoint, the low-cost forward genetic screening workflow established here provides an economical and efficient strategy for dissecting the genetic networks governing organelle morphology and homeostasis and can be readily extended to other subcellular phenotypes. As a genetically tractable eukaryotic model, fission yeast offers a powerful and cost-efficient system for uncovering conserved cellular mechanisms that are directly relevant to higher eukaryotes [36]. In summary, our work identifies the HOPS complex as a novel regulator of LD morphology in fission yeast and delineates a genetic pathway that links vacuolar integrity, Vps13-dependent lipid transport, and LD shape control. These findings broaden our understanding of the functional crosstalk between the endolysosomal system and LDs and underscore membrane contact sites and non-vesicular lipid transport as key determinants of LD homeostasis. Further biochemical and cell biological studies will be needed to identify the specific lipid species transferred by Vps1301 and Vps1302 at LD–vacuole interfaces and to clarify how HOPS-dependent vacuolar organization modulates the activity of these lipid transporters.

## 5. Conclusions

Through a microscopic observation-based forward genetic screen, followed by mutation identification by next-generation sequencing, in the fission yeast *Schizosaccharomyces pombe*, we demonstrate a previously unrecognized function of the HOPS tethering complex in regulating lipid droplet (LD) morphology. Mutations in genes encoding HOPS complex subunits and their Rab GTPase partner Ypt7 were identified as causal determinants of the aberrant LD morphology. Disruption of the HOPS complex resulted in concomitant vacuolar fragmentation and severely impaired the degradation of LD surface protein, but multiple independent lines of evidence indicated that the LD morphological defect does not arise as a secondary consequence of defective lipophagic degradation. Our growth-based suppressor screening found that loss of Ins1, a regulator of sterol biosynthesis, partially rescues the growth defect of the *Δvps11* mutant at restrictive temperature but failed to restore the abnormal LD morphology, indicating that these two phenotypes are regulated via distinct downstream mechanisms; in parallel, a forward genetic screen based on visual observation found that loss-of-function mutations in *vps1302*, as well as deletion of either *vps1301* or *vps1302*, suppress the LD morphological phenotype in *Δvps11* without rescuing the defect of the degradation of LD surface protein. Collectively, our findings describe a novel genetic pathway linking vacuolar integrity, inter-organelle lipid transport, and LD homeostasis. Our work expands current understanding of how the endolysosomal system and LDs interact with each other. It also provides a cost-effective forward genetic strategy for studying organelle morphology and its regulation in fission yeast.

## Figures and Tables

**Figure 1:**
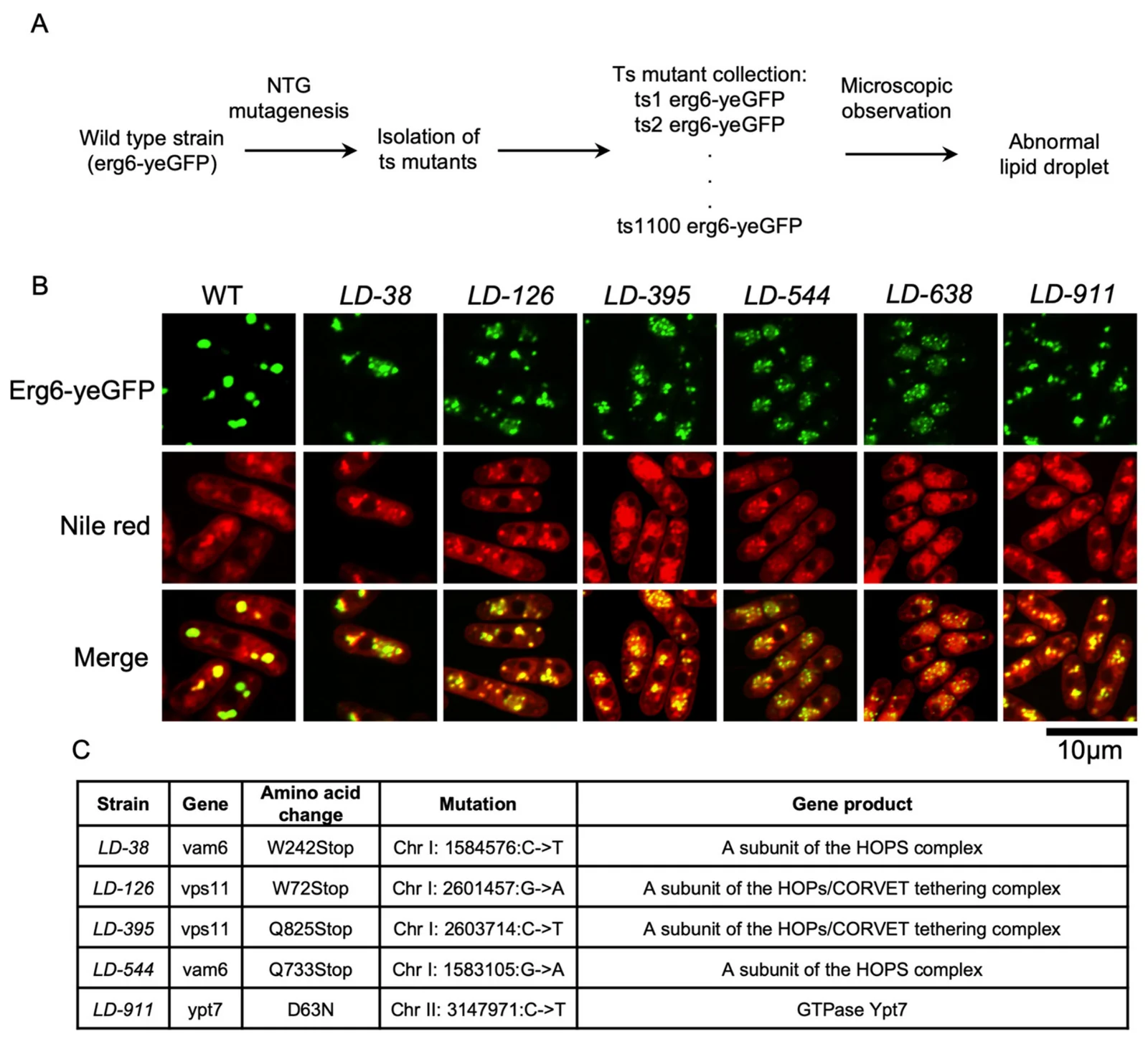
Isolation of mutants with abnormal lipid droplet morphology and mutation identification. (**A**) A flowchart to isolate ts mutants with abnormal lipid droplet morphology. Mutant cells cultured at log phase in rich YES liquid medium at permissive temperature (30 °C) were shifted to restrictive temperature 37 °C for 2 h. Then, cells were collected and observed by fluorescence microscopy. Note: The cells used for the following microscopic observation were all cultured at permissive temperature 30 °C. (**B**) Six mutants with abnormal lipid droplet morphology (indicated by Erg6-yeGFP signals) were cultured at the permissive temperature of 30 °C and then were observed by fluorescence microscopy. Nile red was used to stain cellular lipids. (**C**) The responsible mutations in the mutants with abnormal lipid droplet morphology identified from next-generation sequencing results.

**Figure 2:**
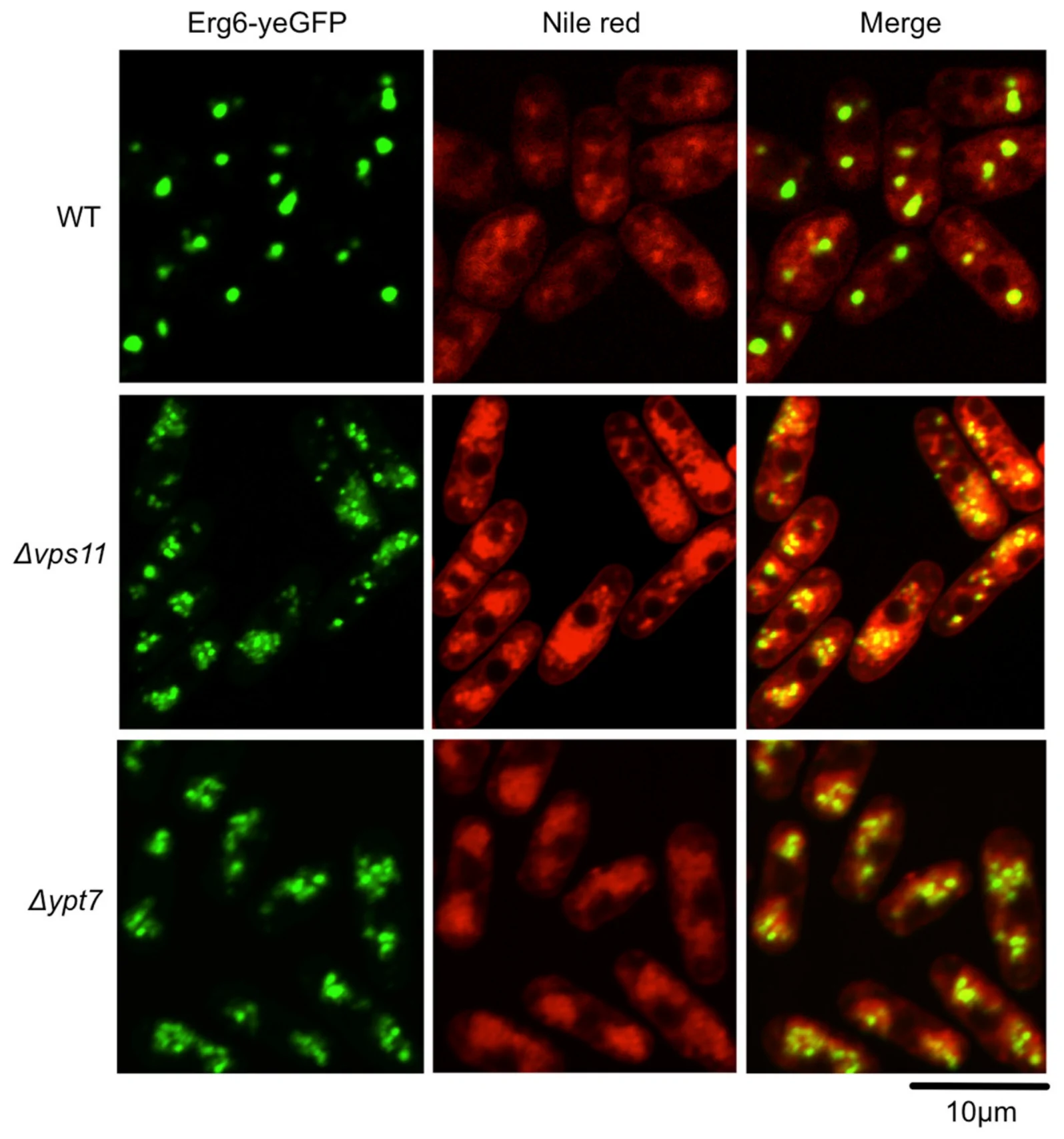
The abnormal lipid droplet morphology was observed in HOPS mutants. Cells of wild type, *Δvps11*, and *Δypt7* were grown to mid-logarithmic phase in rich YES liquid medium at permissive temperature, and lipid droplets (indicated by Erg6-yeGFP signals) were visualized by fluorescence microscopy. Nile red was used to stain cellular lipids.

**Figure 3:**
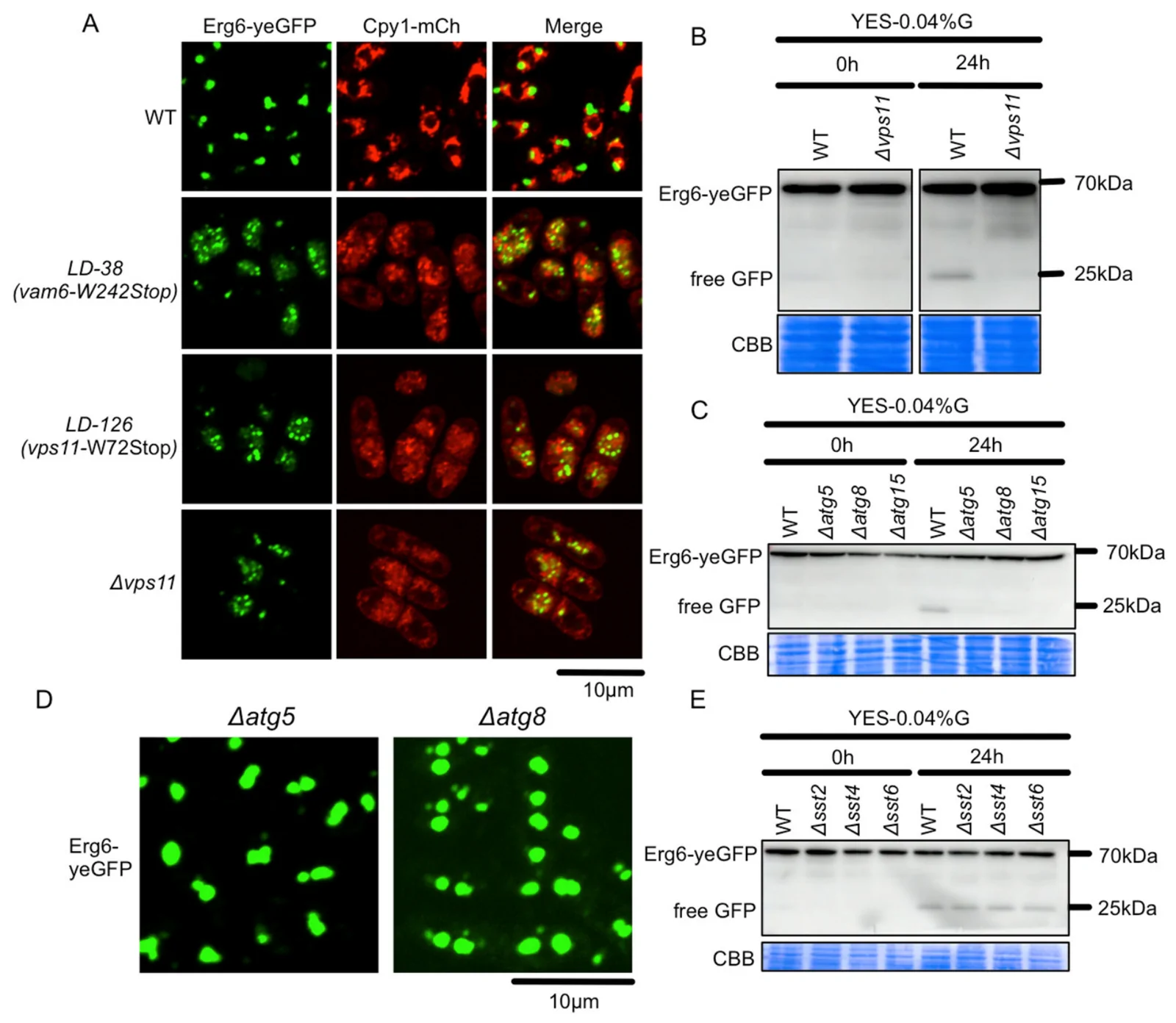
Vacuolar fragmentation and protein degradation defects in HOPS mutants. (**A**) In addition to the abnormal lipid droplet morphology, vacuoles (indicated by Cpy1-mCherry signals) were fragmented in the HOPS mutants when cultured in rich YES medium at permissive temperature 30 °C. (**B**) Glucose starvation (by shifting cells cultured in normal YES medium to YES medium with low glucose concentration (0.04%)) activated the degradation of LD surface protein Erg6 (indicated by the free GFP band detected by the anti-GFP antibody) in wild type and deletion of *vps11* blocked the degradation of Erg6 (disappearance of the free GFP band) dramatically. Coomassie Brilliant Blue (CBB) staining was shown as a loading control. (**C**) The degradation of LD surface protein Erg6 was blocked dramatically in autophagy mutants (*Δatg5*, *Δatg8*, and *Δatg15*) after glucose starvation. (**D**) The lipid droplet morphology in *Δatg5* and *Δatg8* was similar to that in wild type in rich YES medium at permissive temperature. (**E**) The degradation of LD surface protein Erg6 in mutants of the ESCRT pathway (*Δsst2*, *Δsst4*, and *Δsst6*) after glucose starvation was similar to that in wild type.

**Figure 4:**
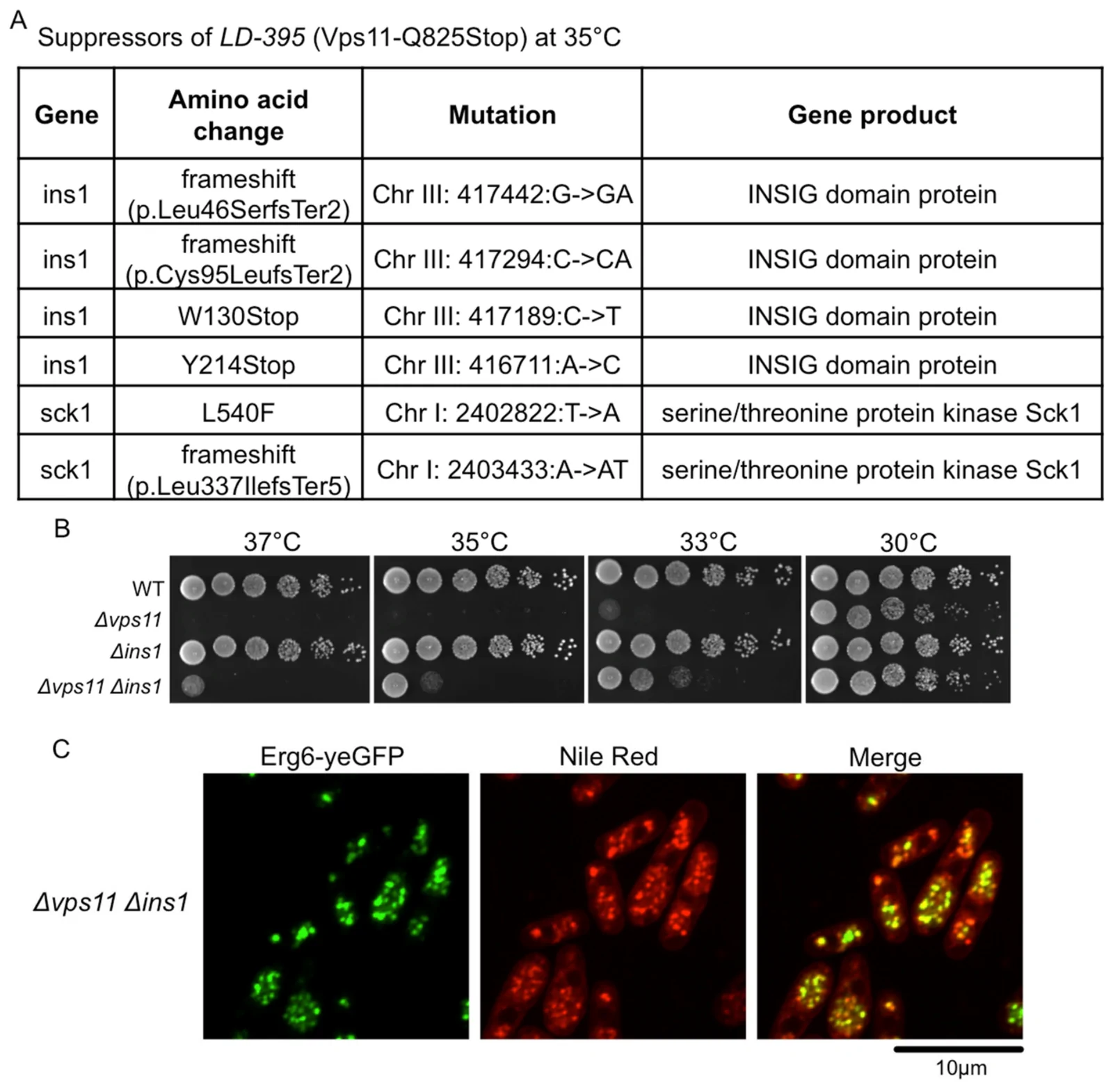
Suppression of *vps11* by *ins1*. (**A**) Spontaneous mutations that could rescue the temperature sensitivity of the *LD-395* mutant. The *LD-395* mutant could not form colony at 35 °C on rich YPD agar plate. Colonies formed at restrictive temperature indicated that an additional mutation that could rescue the growth defect of the *LD-395* mutant was introduced into the mutant during the cell cycle. Next-generation sequencing followed by bioinformatics analysis was applied to identify the suppressor mutations. (**B**) Confirmation of the genetic suppression of the *Δvps11* by *Δins1*. (**C**) Lipid droplet morphology in the *Δvps11 Δins1* double mutant in rich YES liquid medium at permissive temperature.

**Figure 5:**
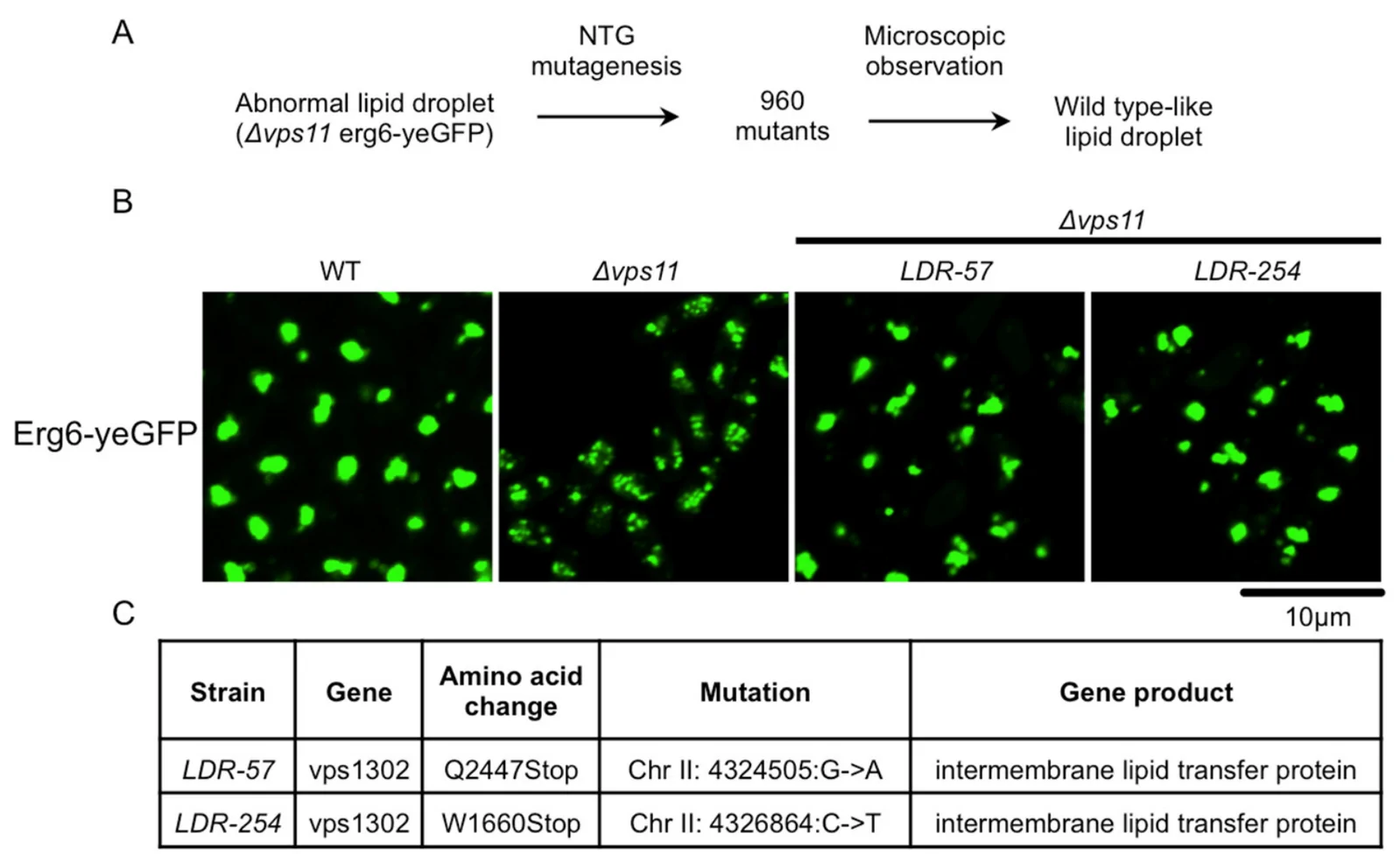
The abnormal lipid droplet morphology in *Δvps11* was rescued by mutations in *vps1302*. (**A**) A flowchart to isolate mutations that could rescue the abnormal lipid droplet morphology in *Δvps11* by observing Erg6-yeGFP signals by fluorescence microscopy. (**B**) Two mutants (*LDR-57* and *LDR-254*) that could rescue the abnormal lipid droplet morphology in *Δvps11* were obtained. The cells were cultured in rich YES liquid medium at permissive temperature. (**C**) The responsible mutations in the *LDR-57* and *LDR-254* mutants identified from the next-generation sequencing results.

**Figure 6:**
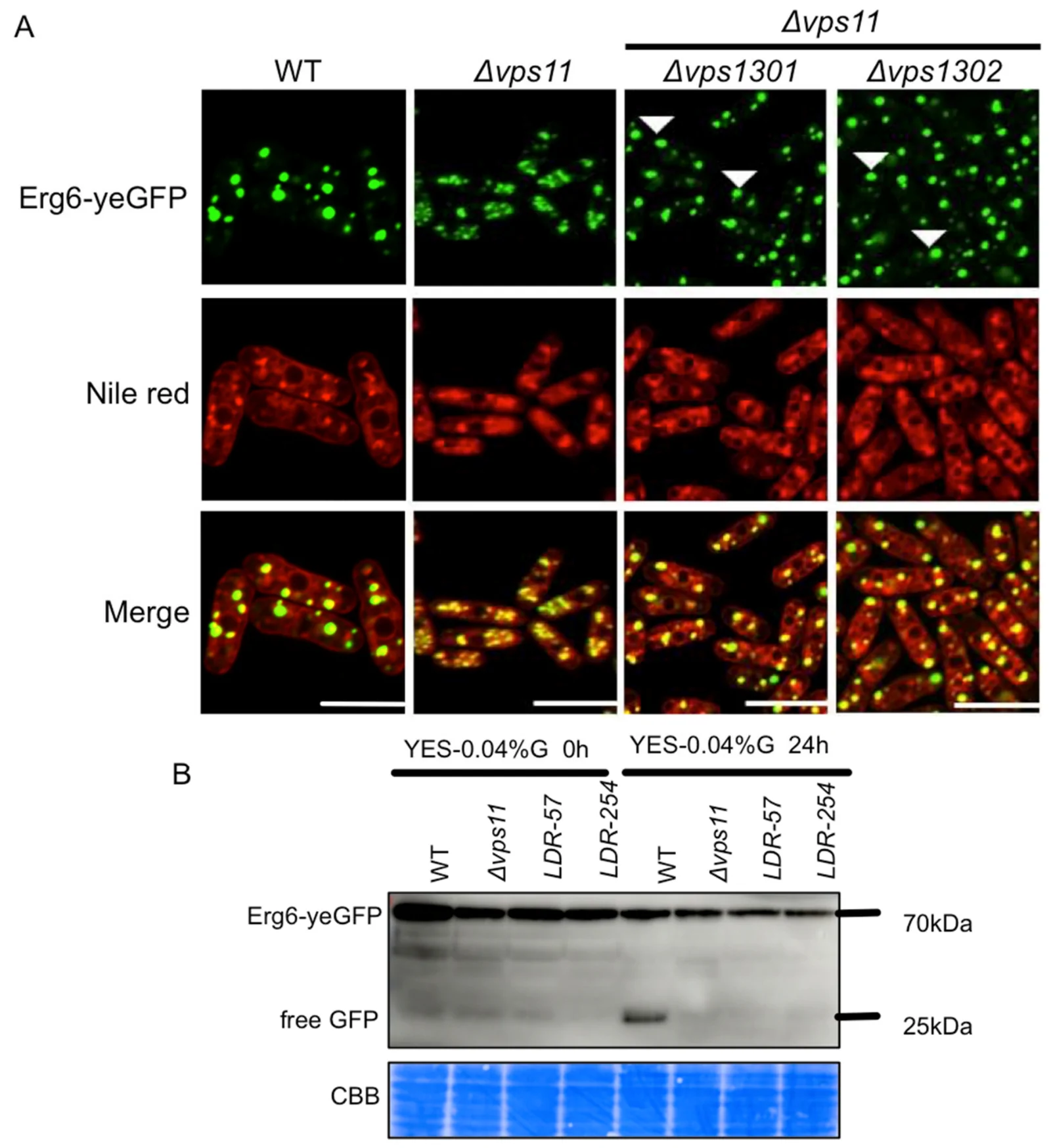
Deletion of *vps1301* or *vps1302* rescued the abnormal lipid droplet morphology in the *Δvps11* mutant. (**A**) Deletion of *vps1301* or *vps1302* rescued the abnormal lipid droplet morphology in the *Δvps11* mutant as well. Cells were cultured in rich YES liquid medium at permissive temperature. (**B**) The defect of the degradation of LD surface protein Erg6 in the *Δvps11* mutant could not be rescued by the *vps1302* mutations in the *LDR-57* and *LDR-254* mutants. The degradation of LD surface protein, Erg6, was indicated by the free GFP band detected by the anti-GFP antibody in immunoblotting. Cells were cultured in rich YES liquid medium and then shifted to YES liquid medium with low glucose concentration (0.04% glucose).

## Data Availability

The next-generation sequencing data reported in this paper have been deposited in the National Center for Biotechnology Information BioProject database with accession no. PRJNA1529342.

## Supplementary Materials

The following supporting information can be downloaded at: https://www.mdpi.com/article/10.3390/cells15191724/s1, Figure S1: The lipid droplet morphology in the *Δvps11 Δatg5* and *Δvps11 Δatg8 double* mutants. Cells were cultured in rich YES liquid medium at the permissive temperature of 30 °C and then were observed by fluorescence microscopy; Figure S2: Lipid droplet morphology in *Δsst* mutants and *Δvps11 Δsst* double mutants. (A and B) Cells of *Δsst* mutants (*Δsst2, Δsst4, Δsst6*) and *Δvps11 Δsst* double mutants (*Δvps11 Δsst2*, *Δvps11 Δsst4*, *Δvps11 Δsst6*) were cultured in rich YES liquid medium at the permissive temperature of 30°C and then were observed by fluorescence microscopy; Figure S3: Lipid droplet morphology in *Δvps1301* and *Δvps1302* mutants. Cells were cultured in rich YES liquid medium at the permissive temperature of 30°C and then were observed by fluorescence microscopy; Table S1: Mixture of F1 progenies of *LD-38* with abnormal lipid morphology (Mutations with frequency ≥ 50% were exhibited); Table S2: Mixture of F1 progenies of *LD-126* with abnormal lipid morphology (Mutations with frequency ≥ 50% were exhibited); Table S3: Mixture of F1 progenies of *LD-395* with abnormal lipid morphology (Mutations with frequency ≥ 50% were exhibited); Table S4: Mixture of F1 progenies of *LD-544* with abnormal lipid morphology (Mutations with frequency ≥ 50% were exhibited); Table S5: Mixture of F1 progenies of *LD-911* with abnormal lipid morphology (Mutations with frequency ≥ 50% were exhibited); Table S6: Mixture of F5 progenies with abnormal lipid morphology of *LD-38*, *LD-126*, *LD-198* (*LD-38*: 20%, *LD-126*: 35%, *LD-198*: 45%); Table S7: Mixture of F5 progenies with abnormal lipid morphology of *LD-421*, *LD-544* (*LD-421*: 30%, *LD-544*: 70%); Table S8: Mixture of F5 progenies with abnormal lipid morphology of *LD-638*, *LD-911* (*LD-638*: 30%, *LD-911*: 70%); Table S9: Mixture of F5 progenies with abnormal lipid morphology of *LD-395*, *LD-196* (*LD-395*: 30%, *LD-196*: 70%); Table S10: Mixture of F1 progenies of *LDR-57* with normal lipid morphology (Mutations with frequency ≥ 50% were exhibited); Table S11: Mixture of F1 progenies of *LDR-254* with normal lipid morphology (Mutations with frequency ≥ 50% were exhibited).
