# Supplementary material for: HOPS and Vps13 Have Antagonistic Roles in Regulating Lipid Droplet Morphology
Source: Cells. 2026 Sep 22;15(19):1724. doi: 10.3390/cells15191724 (PMC13664313; doi:10.3390/cells15191724)
Supplement: Supplementary file 1 [file cells-15-01724-s001.zip › cells-4539706-supplementary figures.pdf]

Supplementary figures

HOPS and Vps13 Have Antagonistic Roles in Regulating Lipid Droplet Morphology

Jie Wang, Saidaiguli Abulimiti, Jingping Chen, Xinping Zheng, Wanjing Chen, Yang Lei and Xingya Xu *

Institute of Future Agriculture, Northwest A&F University, Yangling, Yangling 712100, China.

wangjie0202@nwafu.edu.cn (J.W.); 990917@nwafu.edu.cn (S.A.); 2025051345@nwafu.edu.cn (J.C.); zxp2025@nwafu.edu.cn (X.Z.); linsiran@nwafu.edu.cn (W.C.); 2023060716@nwafu.edu.cn (Y.L.)

*Correspondence: Xingya Xu (xuxingya@nwafu.edu.cn)

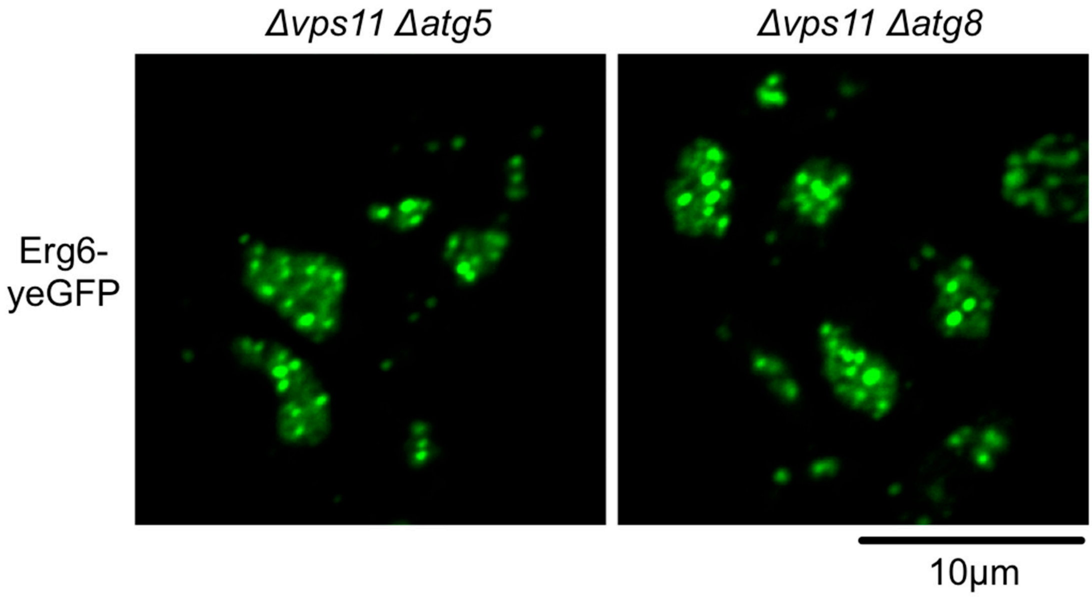

Figure S1. The lipid droplet morphology in the *Δvps11 Δatg5* and *Δvps11 Δatg8* double mutants. Cells were cultured in rich YES liquid medium at the permissive temperature of 30°C and then were observed by fluorescence microscopy.

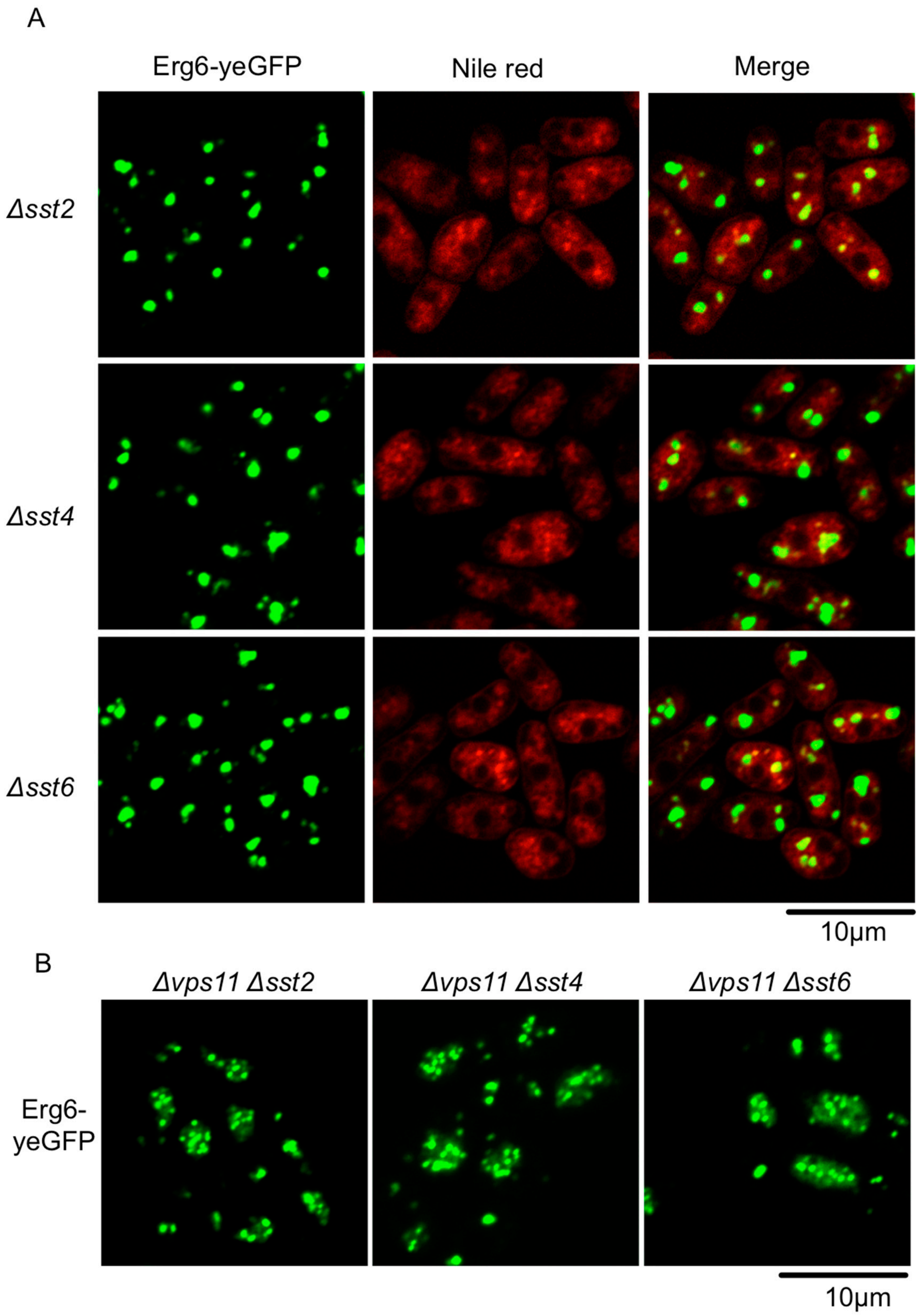

Figure S2. Lipid droplet morphology in *Δsst* mutants and *Δvps11 Δsst* double mutants. (A and B) Cells of *Δsst* mutants *(Δsst2, Δsst4, Δsst6)* and *Δvps11 Δsst* double mutants (*Δvps11 Δsst2, Δvps11 Δsst4, Δvps11 Δsst6*) were cultured in rich YES liquid medium at the permissive temperature of 30°C and then were observed by fluorescence microscopy.

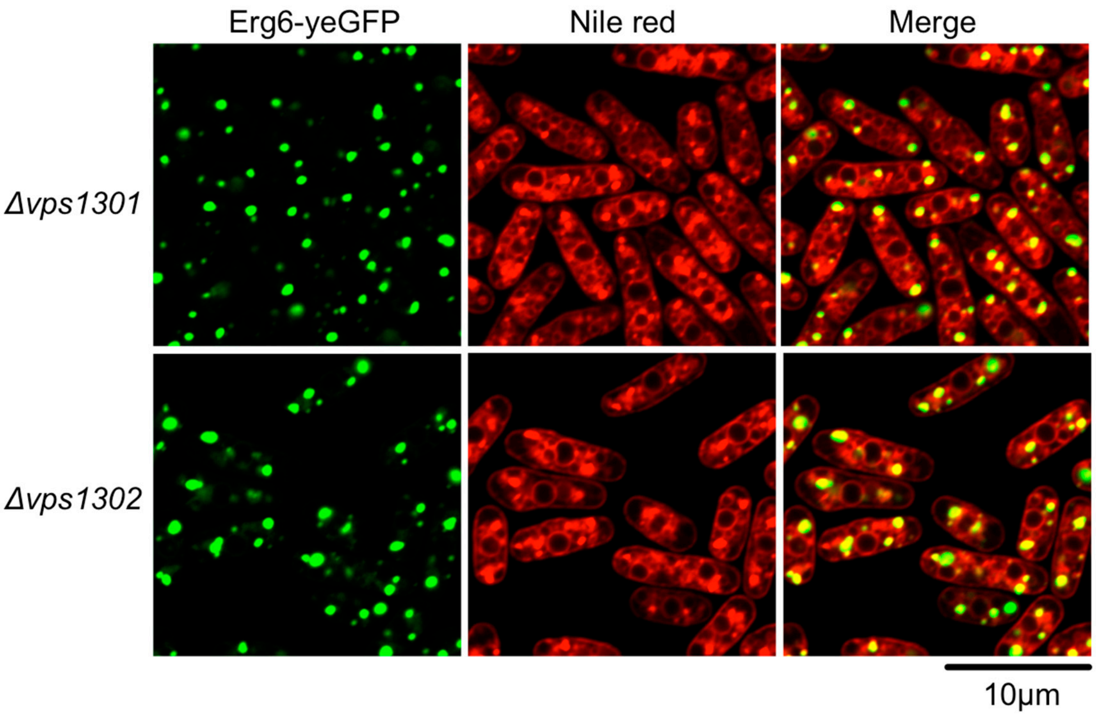

Figure S3. Lipid droplet morphology in *Δvps1301* and *Δvps1302* mutants. Cells were cultured in rich YES liquid medium at the permissive temperature of 30°C and then were observed by fluorescence microscopy.
